# "Motoring" for Health

**Published:** 1902-04-05

**Authors:** 


### "Motoring" for Health.

To a large section of the community the idea of a holiday without plenty of exercise is absurd. In practice there is some truth in this view of the matter—at any rate it is often by no means easy in the case of a patient who is debarred from active muscular exertion to lay down a course which shall give him what he wants, and what he may fairly expect out of his holiday, without at the same time involving a degree of effort which is likely to be injurious to him. So completely indeed have the dangers of the ordinary holiday seemed in some cases to overbalance the advantages to be derived from it that physicians have not hesitated to advise such patients to take a course of "rest cure," or even to throw up business and go to bed, rather than run the risks involved in the rush and tumble of holiday pleasure seeking. And in cases of serious disease this may be the best that one can do. Still there is much in a holiday, in the old sense of the term, which cannot be obtained from mere rest, and it becomes a matter of interest to inquire in what manner one can best obtain the good without the evil for those cases in which muscular effort is contra-indicated. It must be borne in mind that such cases do not exclusively occur among people who would consider themselves invalids. To that great multitude whose daily occupation is of a sedentary nature the good which is derived from holidays is rather on the nervous than the muscular side, and although, if the holiday were a long one, muscular training might well form a part of the régime, one can hardly doubt, especially in regard to people over fifty, that the muscular effort expended in obtaining the necessary change for the nervous system often takes away much of the benefit of a holiday, and sometimes throws the balance quite over to the other side. The excretory organs of a man, even of a healthy man, who leads a sedentary life are not always equal to much more than their ordinary daily work, and when such a person begins his holiday by doubling, or, as is often the case, by far more than doubling, his accustomed exercise his tissues become overcharged with waste products and his nervous system, instead of being recuperated by the fresh air and change of scene, is merely numbed and fagged by the poisonous products of muscular fatigue. It is true enough that a day spent in Alpine climbing is a far greater holiday than a day spent in walking on the flat; that a run on a bicycle is a better mental stimulant than wheeling one's baby in a perambulator; that a day with the hounds gives a far greater fillip to a jaded nervous system than any amount of jogging along the high road; and many people have confused the issue by regarding the greater effort which these things involve as the cause of greater benefit which is derived from them. Far more likely is it that their good influence is due to the concentration of the mind upon topics which are entirely different from those upon which it is fixed in the work of daily life. It is commonly and very truly said that the essence of holiday lies in change. But it is very seldom recognised how difficult it is to turn the mind out of the ordinary rut. One walks along the road—thinking; one takes the ordinary invalid drive—thinking; one throws in one's lot with the tourist and sees many sights, but one's mind goes on in the old ruts, excited for a moment, perhaps, by some new thing, but always returning to its old occupation. This, however, is quite impossible when clambering up a precipice or crossing a crevasse; it is out of the question when negotiating slippery corners on a bicycle, or when jumping hedges or seeking short cuts across the field, and we venture to say that in many of the forms of holiday the benefit of which has been falsely attributed to exercise of muscle, the real cause of their good effects is to be sought in the concentrated exercise of mind on totally new topics. In seeking a good form of mental holiday for those to whom we do not wish to order undue muscular exertion, we must look then for some employment which shall for the time being as fully wipe out all traces of the study and the office as is the case in Alpine climbing or in following the hounds, and yet shall be muscularly easy. For this purpose we believe that the motor car offers many advantages. Our attention is drawn to this subject by an article by "A Physician" in a recent number of *Motoring Illustrated*. He says that he has seen great benefit from holidays spent in motor-driving. "The attendant mental exhilaration, the little pleasurable excitement in the necessary mechanical skill in driving, the draughts of pure fresh air, the alternating influences of hill and valley, the swaying movements bringing muscles into play passively and without exertion, the æsthetic delights to brain and eye of changing scenery—all these are recuperative, tranquillising, and eminently enjoyable." No one can doubt that a holiday on a motor—that is if the motor behaves itself—fulfils the conditions which we have laid down for a holiday without effort. But the motorist must drive himself, for the very essence of mental holiday lies in the mind being forced into new grooves.

